# Host microbiome associated low intestinal acetate correlates with progressive NLRP3-dependent hepatic-immunotoxicity in early life microcystin-LR exposure

**DOI:** 10.1186/s40360-023-00721-7

**Published:** 2023-12-13

**Authors:** Madhura More, Somdatta Chatterjee, Punnag Saha, Dipro Bose, Ayushi Trivedi, Subhajit Roy, Saurabh Chatterjee

**Affiliations:** 1 Environmental Health and Disease Laboratory, Department of Environmental and Occupational Health, Program in Public Health, University of California – Irvine, 92697 Irvine, CA USA; 2 Toxicology Core, NIEHS Center for Oceans and Human Health on Climate Change Interactions, Department of Environmental and Occupational Health, Program in Public Health, University of California – Irvine, 92697 Irvine, CA USA; 3 Division of Infectious Disease, Department of Medicine, UCI School of Medicine, University of California – Irvine, 92697 Irvine, CA USA; 4 IGeneX Inc-Reference Laboratory, 95035 Miltipas, CA USA

**Keywords:** Microcystin, Microbiome, Acetate, NLRP3, GPR43

## Abstract

**Background:**

Microcystins (MCs), potent hepatotoxins pose a significant health risk to humans, particularly children, who are more vulnerable due to higher water intake and increased exposure during recreational activities.

**Methods:**

Here, we investigated the role of host microbiome-linked acetate in modulating inflammation caused by early-life exposure to the cyanotoxin Microcystin-LR (MC-LR) in a juvenile mice model.

**Results:**

Our study revealed that early-life MC-LR exposure disrupted the gut microbiome, leading to a depletion of key acetate-producing bacteria and decreased luminal acetate concentration. Consequently, the dysbiosis hindered the establishment of a gut homeostatic microenvironment and disrupted gut barrier function. The NOD-like receptor family pyrin domain – containing 3 (NLRP3) inflammasome, a key player in MC-induced hepatoxicity emerged as a central player in this process, with acetate supplementation effectively preventing NLRP3 inflammasome activation, attenuating hepatic inflammation, and decreasing pro-inflammatory cytokine production. To elucidate the mechanism underlying the association between early-life MC-LR exposure and the progression of metabolic dysfunction associated steatotic liver disease (MASLD), we investigated the role of acetate binding to its receptor -G-protein coupled receptor 43 (GPR43) on NLRP3 inflammasome activation. Our results demonstrated that acetate-GPR43 signaling was crucial for decreasing NLRP3 protein levels and inhibiting NLRP3 inflammasome assembly. Further, acetate-induced decrease in NLRP3 protein levels was likely mediated through proteasomal degradation rather than autophagy. Overall, our findings underscore the significance of a healthy gut microbiome and its metabolites, particularly acetate, in the progression of hepatotoxicity induced by early life toxin exposure, crucial for MASLD progression.

**Conclusions:**

This study highlights potential therapeutic targets in gut dysbiosis and NLRP3 inflammasome activation for mitigating toxin-associated inflammatory liver diseases.

**Supplementary Information:**

The online version contains supplementary material available at 10.1186/s40360-023-00721-7.

## Introduction

Microcystins (MCs) are a class of potent hepatotoxins produced by cyanobacterial strains like *Anabaena*, *Hapalosiphon*, *Microcystis*, *Nostoc*, *Planktothrix*, and *Phormidium* [1, 2]. Microcystin-LR (MC-LR) is the predominant form of MCs found in fresh and brackish waters. It is also the most toxic and commonly studied congener of MC. Exposure to MC-LR and other MCs commonly occurs via drinking water although there are other routes of exposure like dermal and inhalation of aerosolized MC particles [3–5]. Children are particularly vulnerable to health effects from exposure to MC-LR via drinking water as they have a higher water intake per body weight compared to adults [6]. Also, children are at an increased risk of exposure to MCs from recreational activities around water bodies. Further, infants and children are especially susceptible to toxic insults as their liver is still undergoing development. At around 2 years from birth, the human liver is completely developed and functional [7].

The gut microbiome is highly vulnerable to toxin exposure during early life, as it is not fully established and only stabilizes around three years of age [8–11]. The colonization of the gut by Bacteroidetes is a crucial early step in microbiome establishment, commencing after weaning when the intake of dietary fiber also increases [12]. The breakdown of complex polysaccharides into simple sugars and short-chain fatty acids (SCFAs), such as acetate through the Wood–Ljungdahl pathway and pyruvate decarboxylation to acetyl–CoA pathways, plays a key role in cross-feeding and the establishment of beneficial bacteria in the gut microbiome [13–15]. A toxic insult during early life that disrupts the colonization by beneficial bacteria can have enduring consequences on gut health and overall organ homeostasis in adulthood through various Gut–Organ Axis pathways [13, 16–20].

Previous studies from our research group have shown that dysbiosis associated with non-alcoholic fatty liver disease (NAFLD) [currently termed as metabolic dysfunction-associated steatotic liver disease (MASLD)] like conditions following MC-LR exposure results from the activation of nucleotide-binding domain-like receptor protein 3 (NLRP3) inflammasome [21, 22]. Also, early life exposure to MC-LR led to the potentiation of MASLD in adulthood which was further exacerbated by the consumption of a high-fat diet and this effect was absent in mice that lacked NLRP3 [23]. Thus, activation of the NLRP3 inflammasome is key to the development of MC-LR induced pathological symptoms of MASLD in adult life.

The NLRP3 inflammasome is a pattern-recognition multiprotein receptor complex, composed of the pro-caspase 1 effector protein, the adaptor protein caspase-recruitment domain apoptosis-associated speck-like protein 2 (ASC2), and the cytoplasmic receptor NLRP3, commonly known as the inflammasome sensor molecule [24]. It is important for defense against pathogens and the maintenance of homeostasis. The canonical activation of NLRP3 inflammasome is a two-step process – priming and assembly. Firstly, Toll–like receptor 4 (TLR4) activation and/or tumor necrosis factor (TNF) signaling leads to priming that includes increased mRNA expression of NLRP3, pro-interleukin-1β and pro-interleukin-18 genes [25–27]. Secondly, recognition of pathogen-associated molecular patterns (PAMPs) and damage-associated molecular patterns (DAMPs) including reactive oxygen species (ROS), mitochondrial dysfunction-related release of mitochondrial DNA and cardiolipin, ion flux changes (K^+^/Cl^−^ efflux, and Ca^2+^ influx), and lysosomal damage can trigger assembly of the NLRP3 inflammasome complex [28–31]. The activation of NLRP3 inflammasome leads to proteolytic activation of interleukin-1β (IL-1β), interleukin-18 (IL-18), and Gasdermin D. Gasdermin D oligomerizes to form a pore complex at the plasma membrane leading to pyroptotic cell death and release of mature IL-1β and IL-18 [32]. Pyroptosis due to NLRP3 inflammasome activation has been noted as a mechanism of MC-LR induced intestinal and hepatotoxicity [33, 34].

Previous studies along with preliminary microbiome analysis for this research work indicated that there was a depletion in key SCFA-producing bacteria and decreased luminal acetate concentration due to early life exposure to MC-LR [35, 36]. Previous literature indicates that SCFAs play a vital role in maintaining gut barrier integrity and preventing the systemic circulation of gut microbiome-derived inflammatory triggers like bacterial lipopolysaccharide, peptidoglycan, flagellin, and nucleic acid variants [14, 37, 38]. Further, SCFA like acetate and butyrate are known to be immunomodulators [39–42]. Higher circulation of PAMPs and DAMPs due to gut leaching is associated with the onset and progression of MASLD pathology to more severe forms like steatohepatitis and liver cirrhosis [20, 23].

Acetate, a SCFA, has been shown to possess anti-inflammatory properties. Acetate has been found to inhibit the production of pro-inflammatory cytokines, such as IL-6 and TNF-α by immune cells [43, 44]. It has been shown to promote the polarization of macrophages towards an anti-inflammatory M2 phenotype at high doses, which produces anti-inflammatory molecules and helps resolve inflammation [45]. Acetate can bind to and activate specific GPRs, including GPR43 and GPR41. GPR43 activation by acetate inhibits the production of pro-inflammatory cytokines in immune cells, contributing to the suppression of inflammation. Acetate binding to its receptor GPR43 leads to attenuation of NLRP3 inflammasome activation by causing NLRP3 protein degradation in bone marrow-derived macrophages [43]. Acetate serves as an energy source for various cells, including immune cells, intestinal epithelial cells, and colonocytes [46]. Thus, acetate promotes the maintenance of a healthy gut barrier. While more research is needed to fully understand the specific anti-inflammatory mechanisms of acetate, as an important SCFA acetate contributes to the overall anti-inflammatory environment in the gut and the body [13].

Taking into consideration the immunomodulatory role of acetate, the depletion of key SCFA-producing bacteria, and lower concentration of acetate in luminal contents due to early life exposure to MC-LR, we were interested in understanding the effect of acetate supplementation on MC-LR related intestinal and hepatic pathophysiology. Therefore, we aimed to explore the role of the SCFA acetate on intestinal and hepatic inflammation in this current study using an established murine model of early-life MC exposure. We also investigated whether oral acetate supplementation prevented the MC-LR induced pro-inflammatory phenotype leading to MASLD–like outcomes in adulthood and the mechanistic role of acetate-mediated attenuation of NLRP3 inflammasome activation. To this end, we used a juvenile mice model primed with MC-LR with or without oral supplementation of SCFA acetate and appropriate control groups. We speculated that the mechanism for such an effect would be linked to the regulation of NLRP3 inflammasome activation.

## Materials and methods

### Materials

Sodium acetate was acquired from Sigma-Aldrich (St. Louis, MO, USA), and MC-LR was purchased from Cayman Chemical Company (Ann Arbor, MI, USA). Primary antibodies against CD68, α-SMA, Claudin2, Occludin, and NLRP3 were acquired from Abcam (Cambridge, MA, USA). Primary antibodies against IL-1β, ASC2, and β-actin were purchased from Santacruz Biotechnology in Dallas, Texas, USA. The Vector Labs (Burlingame, CA, USA) provided the species-specific biotinylated conjugated secondary antibody and the Streptavidin-horseradish peroxidase (Strp-HRP) (Vectastain Elite ABC kit). Thermo Fisher Scientific (Waltham, MA, USA) supplied the fluorescence-conjugated Alexa Fluor secondary antibodies and ProLong Diamond antifade mounting medium with 4′,6-diamidino-2-phenylindole (DAPI). All of the chemicals required for this study were bought from Sigma-Aldrich (St. Louis, MO, USA) unless otherwise stated. AML Laboratories (St. Augustine, Florida, USA) processed the mouse liver and intestinal tissues for paraffin embedding and sectioning into slides. Cosmos ID (Rockville, Maryland, USA) carried out the bacteriome analysis.

### Animals

Male, juvenile, wild-type (WT), pathogen-free C57BL/6J mice were obtained from Jackson Laboratories (Ban Harbor, ME, USA) and used in this work. The Institutional Animal Care and Use Committee (IACUC) and National Institutes of Health (NIH) guidelines for the humane care and use of laboratory animals were strictly followed in all mouse experiments. The animal experimentation techniques for this work were approved by the University of South Carolina in Columbia, South Carolina, and adhered to ARRIVE guidelines.

All mice were placed in a temperature-controlled room (22–24 °C) with a 12-hour light/12-hour dark cycle and they had unlimited access to food and water. After dosing was complete at the age of 10 weeks all mice were euthanized. The primary method of euthanasia was overdose of anesthetic – isoflurane. Inhalation exposure to 4% isoflurane was continued for a minute after respiratory arrest. Euthansia was confirmed by cervical dislocation. Liver and small intestine tissue from each mouse were removed and promptly preserved in 10% neutral buffered formaldehyde (Sigma-Aldrich, St. Louis, MO, USA). Additionally, serum samples were extracted from freshly drawn blood and maintained at a temperature of − 80 °C. Fecal pellets were collected then immediately snap-frozen and kept at − 80 °C for bacteriome analysis.

### Experimental animal model

24 WT mice (4 weeks old, post-weaning) were used in this study, and they were randomly assigned to one of four groups (n = 6 per group): control group (CHOW), MC-LR treated (MC), acetate supplementation followed by MC-LR treated (MC + AC), or solely acetate supplemented (AC). For the whole period of the trial, from 4 to 10 weeks of age, the groups MC + AC and AC were supplemented with acetate at a dose of 2 g/kg via oral gavage. Based on earlier research, this dose of acetate was chosen [47, 48]. The treated group (MC) of mice were dosed with MC-LR [5 µg/kg body weight; dissolved in ethanol and then diluted in Phosphate buffered saline (PBS) for a continuous 2 weeks by oral gavage route [8] whereas the control group (CHOW) of mice got just vehicle (PBS) at the age of 6 weeks. All WT mice received the MC-LR treatment, were given a 4-week rest period to allow them to grow, and were then euthanized at the age of 10 weeks. Based on results from our previous research and preliminary studies, it was determined that the experiments maintain a specified sample size of n = 3 for each treatment group that had a power of 0.08 at an alpha of 0.05.

### Rat primary Kupffer cell culture

Primary rat Kupffer cells were purchased from Cell Biologics, Inc. (Chicago, IL, USA). The cells were maintained in Dulbecco’s modified Eagle’s medium (DMEM) (Catalog number: 11995065 Thermo Fisher Scientific, Rockford, IL, USA) and supplemented with 10% fetal bovine serum (FBS) (Catalog number: F-0500-D, Atlas Biologicals, Fort Collins, CO, USA), 2mM L-Glutamine, 10U/L Penicillin and 100 µg/kg Streptomycin (Gibco, NY, USA) and incubated in a humidified 5% CO_2_ incubator at 37 °C. The cells were plated in 6-well tissue culture plates and were allowed to grow till they reached around 70% confluency. After overnight serum starvation (0.2% FBS) the cells were treated with bafilomycin A1 (40 nM) [47, 48] an autophagy inhibitor, or with a GPR43 antagonist GLPG0974 (0.1 µM) for 1 h before treatment [49]. Cells were then treated with vehicle (Control), MC-LR (20 µM), and acetate (3.5 mM)(Table-1). Acetate concentration for treatment was determined based on serum acetate concentration measured by acetate assay in samples from the CHOW group. Cell pellets were collected to extract total proteins using RIPA buffer.

**Table 1:** Rat Kupffer cells experiment outline

GROUP	INHIBITORS		ACETATE		MC-LR
CONTROL	**-**	1 h at 37 °C	**-**	6 h at 37 °C	**-**
MC	**-**		**-**		**+**
MC + AC	**-**		**+**		**+**
AC	**-**		**+**		**-**
MC + AC + Bafilomycin A1	**+**		**+**		**+**
MC + AC + GLPG0974	**+**		**+**		**+**

### Bacteriome analysis

The detailed methodology for bacteriome analysis is mentioned elsewhere [8, 50]. Briefly, the vendor CosmosID Inc. (Germantown, MD, USA) used fecal pellets from all experimental mice for DNA isolation and preparation of raw reads. The HiSeq X platform was used for whole-genome sequencing, and the taxonomic results were reviewed for barcoding or contamination issues. Bacteriome analysis was completed using the MetaWRAP pipeline, and the reads were trimmed, eliminated, and assembled using Trim Galore, BMTagger 1.1.0, and MegaHit 1.2.9 respectively. The NCBI Bacteria Database was used for mapping the reads and producing a list of taxa and abundances.

## Methods

### Histopathology

Formalin-Fixed paraffin-Embedded (FFPE) liver tissues were cut into 5 μm thick sections. For histological examinations, liver section slides were deparaffinized using a standard protocol.

### Immunohistochemistry

Formalin-fixed, paraffin-embedded liver tissue sections were deparaffinized using standard laboratory protocol. All 5 μm-thick tissue sections were immersed in 100% xylene first, followed by a 1:1 solution of xylene and ethanol, then 100% ethanol, 95% ethanol, 70% ethanol, and 50% ethanol in succession, and finally in deionized water for 3 min each. Epitope retrieval solution and steamer (IHC-World, Woodstock, MD, USA) were used for antigen epitope retrieval of the tissue Sect. 3% H_2_O_2_ solution was used to block the endogenous peroxidase activity for 20 min, followed by serum blocking (5% goat serum, 1 h). Sections were incubated overnight at 4 °C with primary antibodies for CD68, α-SMA, and IL-1β as recommended dilutions (1:300 in blocking buffer) in a humidified chamber. All tissue sections were washed with 1X PBS-T (PBS + 0.05% Tween 20) 3 times. Species-specific biotinylated secondary antibodies (1:250 in blocking buffer) followed by streptavidin-conjugated with horseradish peroxidase (1:200 dilution) were used according to the manufacturer’s standard protocols. Finally, 3,3 diaminobenzidine (DAB) (Sigma-Aldrich) was used as a chromogenic substrate and counter-stained with Mayer’s hematoxylin (Sigma-Aldrich). Tissue sections were washed with 1X PBS-T between the steps. Sections were finally mounted in Simpo mount (GBI Laboratories, Mukilteo, WA) and observed under a 20X objective using an Olympus BX43 microscope (Olympus, America). Morphometric analysis was done using CellSens Software from Olympus America (Center Valley, PA).

### Immunofluorescence and microscopy

Deparaffinization and epitope retrieval procedures of the paraffin-embedded liver and small intestine sections were carried out in an exactly similar manner as mentioned for the immunohistochemistry method. After the epitope retrieval process was completed, all tissue sections were permeabilized using PBS-Tx (PBS + 0.1% Triton X-100) solution for 1 h, followed by blocking with 5% goat serum for 1 h. Following the blocking step, the tissue sections were probed with primary antibodies against Claudin2, Occludin, NLRP3, and ASC2 (1:300 dilution) and kept overnight at 4 °C in a humidified chamber. After that, species-specific anti-IgG secondary antibodies conjugated with Alexa Fluor 488 or 633 from Invitrogen (Rockford, IL, USA) were applied to the Sect. (1:250 dilution). Lastly, ProLong Gold antifade reagent with DAPI (Life Technologies, Carlsbad, CA, USA) was used to mount the tissue sections. All immunofluorescence-stained images for this study were captured by an Olympus BX63 microscope (Olympus America, Center Valley, PA, USA) using the 40× objective. Analyses of all morphometric data were performed using CellSens Software from Olympus America (Center Valley, PA, USA).

### Western blot

Using a 1x RIPA lysis solution containing protease and phosphatase inhibitors, proteins were isolated from liver samples. The BCA assay kit (Thermo Fisher Scientific, Waltham, Massachusetts, USA), was used to measure the extracted tissue protein concentration. 1× NuPAGE™ LDS Sample Buffer (Thermo Fisher Scientific, Waltham, Massachusetts, USA) and 10% β-mercaptoethanol were added to 40 µg of the extracted protein from each liver sample before boiling for 5 min. The liver-extracted protein samples were subsequently separated using a normal SDS-PAGE procedure utilizing Novex 4–12% bis-tris gradient gel. The Trans-Blot Turbo transfer system (Bio-Rad, Hercules, CA, USA) was used to transfer the separated protein bands onto a nitrocellulose membrane following blocking with 3% bovine serum albumin (BSA) for an hour. Blots were incubated with primary antibodies against NLRP3, IL-1β, and β-actin (1:1000 dilution) and stored at 4 °C overnight. Compatible species-specific HRP-conjugated secondary antibodies were applied (1:2500 dilution) to the blots after three washes with 1X TBS-T (Tris-buffered saline + 0.05% Tween 20) buffer. The blots were developed using Pierce ECL Western blotting substrate from Thermo Fisher Scientific, Waltham, Massachusetts, USA. Finally, G: Box Chemi XX6 was used to capture the photographs of the blots. All densitometric analyses were performed using Image J software (NIH, Bethesda, MD, USA).

### Acetate assay

Acetate concentration (mM) in the CHOW and MC groups of mice was quantified using the EnzyChrom™ Acetate Assay Kit from BioAssay Systems (Hayward, CA, USA) following the manufacturer’s standard protocol. Firstly, 10µL standard and test samples were appropriately diluted using the supplied sample diluent and then applied to the designated wells in a clear, flat-bottom, 96-well plate. The working reagent was freshly prepared as per manufacturer’s instructions (90 µL Assay Buffer, 5 µL Enzyme A, 1 µL Enzyme B, 1 µL Dye Reagent and 1 µL ATP per well) and applied to each well. The plate was then incubated at room temperature for 30 min. Absorbance was measured at 570 nm using a microplate reader. The acetate concentration was then calculated using the standard curve.

### Statistical analysis

The GraphPad Prism program (San Diego, CA, USA) was used to perform all statistical analyses for this investigation. For this investigation, all data are presented as the mean ± SEM. For intergroup comparison, unpaired t-tests (two-tailed tests with equal variance) were used, followed by Bonferroni-Dunn post hoc corrections analysis. *p* ≤ 0.05 was regarded as statistically significant for all analyses.

## Results

### Early life exposure to MC-LR led to an altered gut microbiome signature specific to SCFA production in adolescence and decreased intestinal acetate production

Previous studies from our research group reported that MC-LR exposure in adulthood alters the gut microbiome [8, 20, 21]. We wanted to examine the effect of early-life exposure to MC-LR on the murine gut microbiome. To show that early life MC exposure alters acetate producing bacteria and was correlated with intestinal acetate levels, we performed next generation sequencing of fecal pellets and intestinal content of the host. A detailed bacteriome profile was obtained and analyzed.

**Fig. 1:**
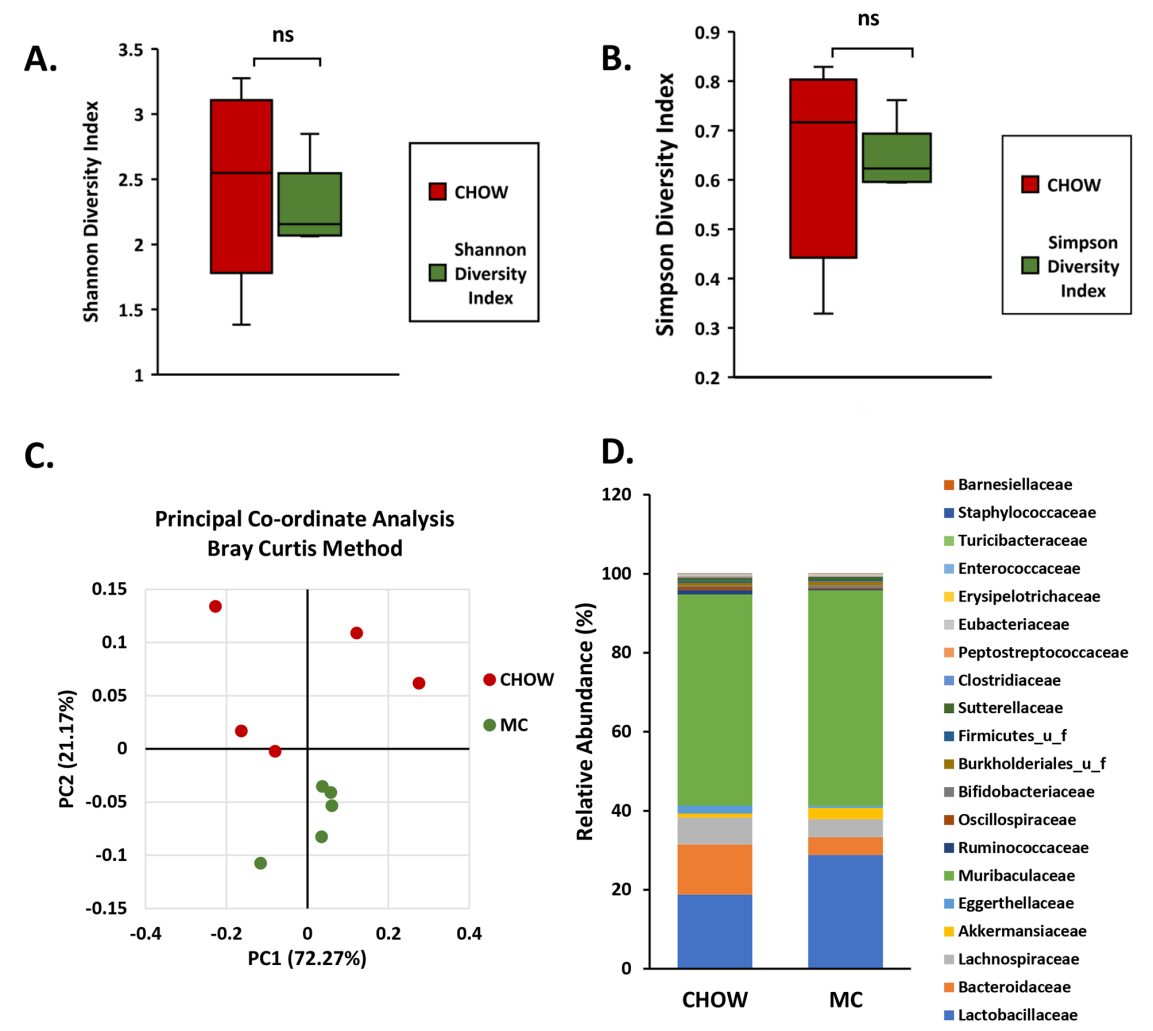
Early life exposure to MC-LR in mice lead to an altered microbiome signature in adolescence. (**A**) Box plot indicating α- diversity index – Shannon diversity of CHOW (mice treated with vehicle only) and MC groups (mice treated with 5 µg/kg MC-LR via oral gavage at 4 weeks of age). (**B**) Box plot indicating α- diversity index – Simpson diversity of CHOW and MC groups. (**C**) Bray–Curtis β-diversity plot in both the CHOW and MC groups. (**D**) The relative abundance of the gut bacteriome at the family level for CHOW and MC groups is presented by group average

We observed that there was a decrease in α-diversity indices of the gut microbiome present in fecal pellets collected from the MC group compared to the CHOW group. However, this decrease was not statistically significant (Fig. 1A and B). As a representation of β-diversity, Principal Co-ordinate Analysis by Bray – Curtis Method was performed. Both CHOW and MC groups formed separate clusters on the β-diversity plot indicating the presence of distinct bacteriome profiles between the two groups (Fig. 1C).

**Fig. 2:**
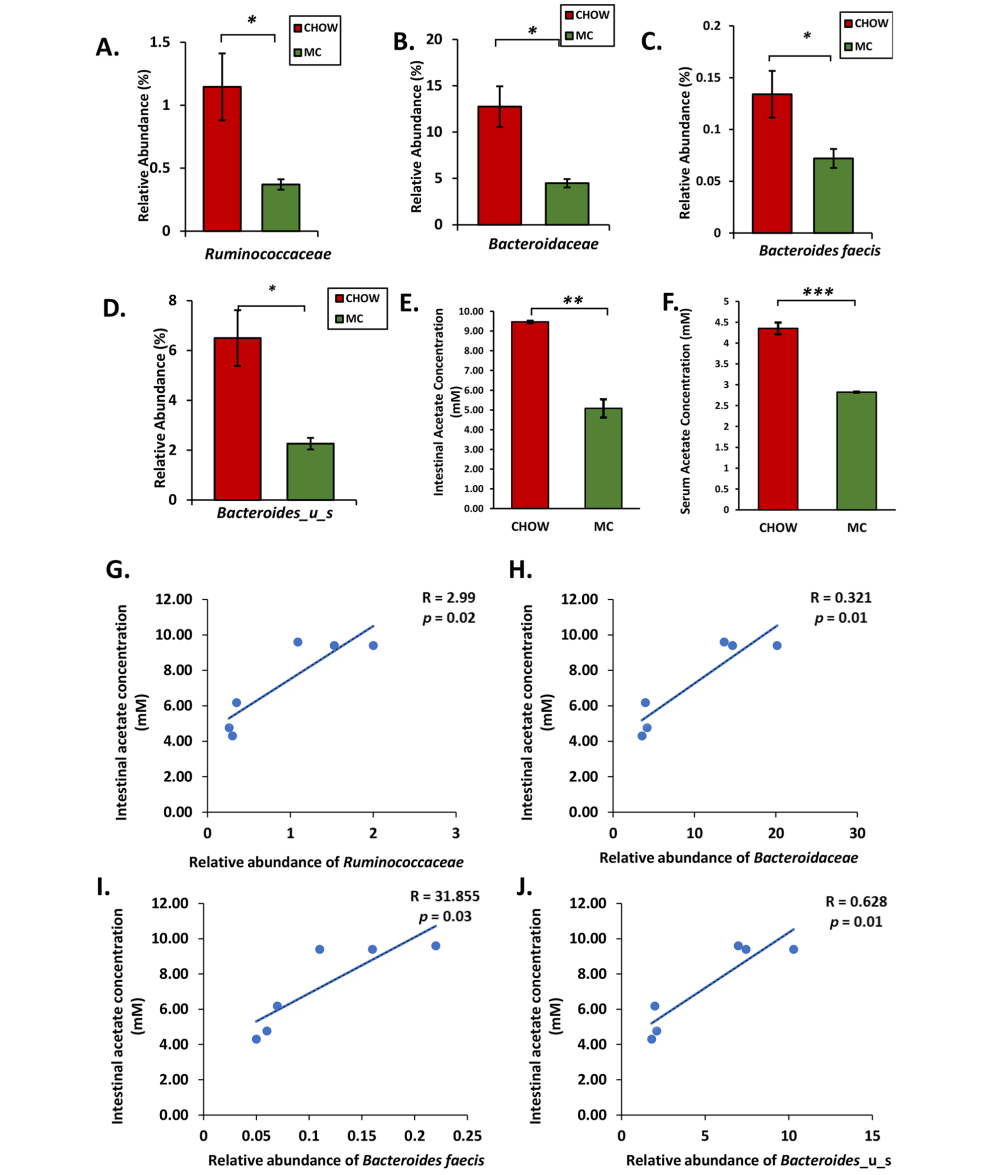
Bar graphs showing the percentage relative abundance of significantly altered bacteria, (**A**) *Ruminococcaceae*, (**B**) *Bacteroidaceae*, (**C**) *Bacteroides faecis*, and (**D**) Bacteroides_u_s. (**E**) Intestinal and (**F**) Serum acetate levels (in mM) of both CHOW and MC mice groups are represented as bar graphs (ns – non significant, * *p* < 0.05, ** *p* < 0.01, *** *p* < 0.001). Data were represented as mean ± SEM and statistical significance was tested using unpaired t-test between the two groups followed by Bonferroni–Dunn post hoc corrections. Correlation plot between intestinal acetate concentration and relative abundances of the bacterial families (**G**) *Ruminococcaceae* and (**H**) *Bacteroidaceae* and bacterial species (**I**) *Bacteroides faecis* and (**J**) Bacteroides_u_s in the gut microbiome

Next, we wanted to determine whether some bacterial families were particularly affected by MC-LR exposure early in life. At the family level, the bacteria belonging to the families *Bacteroidaceae*, *Lachnospiraceae*, *Eggerthellaceae*, *Ruminococcaceae*, *Oscillospiraceae*, *Firmicutes*_u_f, *Clostridiaceae*, *Peptostreptococcaceae*, *Eubacteriaceae*, *Enterococcaceae*, *Turicibacteraceae*, and *Barnesiellaceae* were decreased and the ones belonging to families *Lactobacillaceae*, *Akkermansiaceae*, *Muribacullaceae*, *Bifidobacteriaceae*, *Burkholderiales*_u_f, *Sutterallaceae*, *Erysipelotrichaceae*, and *Staphylococcaceae* increased (Fig. 1D). Out of the 12 bacterial families whose relative abundances decreased in MC group, the families *Ruminococcaceae* (*p* = 0.03, Fig. 2A) and *Bacteroidaceae* (*p* = 0.03, Fig. 2B) are particularly important as they are major producers of SCFA in the gut.

To determine which species of bacteria were differentially enriched between the CHOW and MC groups, we looked at relative abundances of key SCFA-producing bacteria in both CHOW and MC groups respectively. We observed that there was a significant decrease in the relative abundance of *Bacteroides faecis* (*p* = 0.028, Fig. 2C) and *Bacteroides*_u_s (*p* = 0.03, Fig. 2D) in the MC group. Both *Bacteroides faecis* and *Bacteroides_u_s* bacteria belong to the phylum *Bacteroidetes* and are essential SCFA producers that play varied roles in the gut ecosystem like protection from invading pathogens and breaking down complex carbohydrates like glycans to provide nutrition for other gut commensals [12]. In humans during infancy (equivalent to 4 to 8 weeks in mice) there is an increase in the population of bacteria belonging to the family *Bacteroidaceae* due to increased consumption of fibers and acetate is the major SCFA produced during this time. *Bacteroides* and SCFA acetate is important for the establishment of a healthy gut microbiome in adulthood [12]. Therefore, the decreased relative abundance of *Bacteroides* due to early-life MC exposure can lead to disturbed intestinal homeostasis which might be persistent in adulthood.

Next, we wanted to examine whether the decreased abundance of important SCFA-producing bacterial families and species led to a change in intestinal acetate production. Indeed, we detected a significant decrease in the acetate concentration in the intestinal lumen in the MC group compared to the CHOW group as measured by acetate assay (*p* = 0.002, Fig. 2E). This depletion in acetate concentration in the intestinal lumen also led to decreased uptake of acetate in circulation reflected by a decreased serum acetate concentration in the MC group compared to the CHOW group (*p* = 0.001, Fig. 2F). Intestinal acetate concentration also correlated positively with the relative abundance of *Ruminococcaceae* (Fig. 2G), *Bacteroidaceae* (Fig. 2H), *Bacteroides Faecis* (Fig. 2I) and *Bacteroides spp* (Fig. 2J) suggesting a decrease in the abundance of acetate producing bacteria led to a decrease in the acetate concentration in the intestinal lumen.

Therefore, early-life exposure to MC-LR in juvenile mice led to gut dysbiosis, and decreased abundance of acetate-producing gut residents which led to depleted acetate production in the intestinal lumen.

### Gut dysbiosis and the consequent decrease in acetate production in the intestinal lumen were associated with poor gut barrier integrity

A healthy gut microbiome and its metabolites are necessary for maintaining gut barrier integrity and preventing the translocation of bacterial cells, metabolites, and toxins into circulation [14, 37, 38]. Claudin 2 and Occludin are tight junction proteins that are necessary for the maintenance of gut epithelial barrier integrity.

**Fig. 3:**
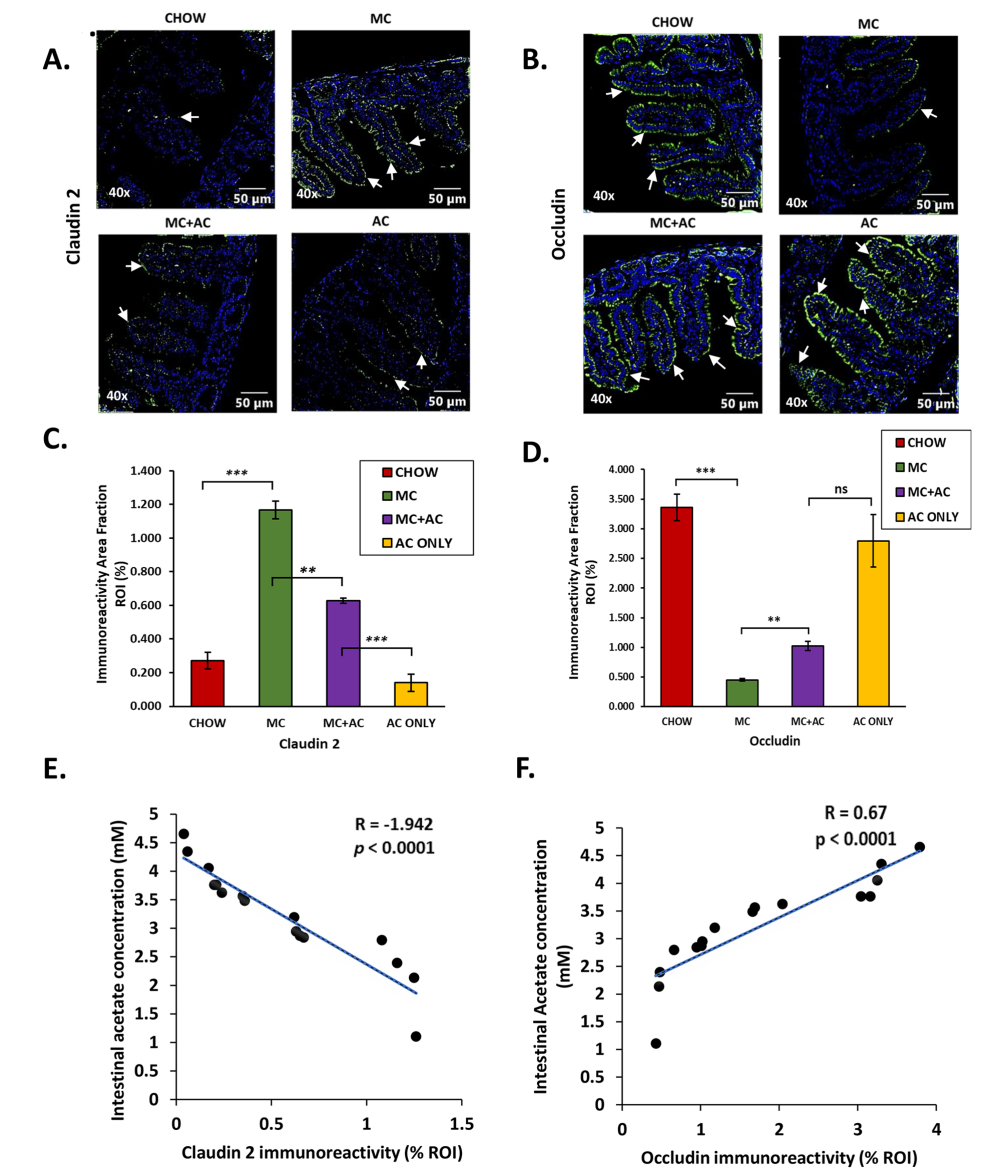
Representative immunofluorescence images of (**A**) Claudin-2 and (**B**) Occludin immunoreactivity (green) from CHOW, MC, MC + AC and AC only groups. Morphometric analysis (calculated as %ROI) of (**C**) Claudin-2, and (**D**) Occludin immunoreactivity where Y-axis represents % positive immunoreactive area (% ROI) (n = 3; mean value taken from three separate microscopic fields). Data were represented as mean ± SEM and statistical significance was tested using unpaired t-test between the two groups (ns – non significant, * *p* < 0.05, ** *p* < 0.01, *** *p* < 0.001), followed by Bonferroni Dunn Post hoc corrections. Correlation plot between intestinal acetate concentration and intestinal (**E**) Claudin-2, and (**F**) Occludin levels

We wanted to evaluate the effect of early life exposure to MC-LR on gut barrier integrity in mice and we observed a marked alteration in the protein levels of Claudin 2 and Occludin by immunofluorescence method. Morphometric analysis of immunostained small intestine sections revealed that early life exposure to MC-LR led to a significant increase in immunoreactivity of Claudin 2 in the MC group compared to the CHOW group (*p* < 0.001 Fig. 3A and C) and a corresponding decrease in immunoreactivity of Occludin in the CHOW group compared to the MC group (*p* < 0.001 Fig. 3B and D). However, we also observed that the immunoreactivity of Claudin 2 was decreased in the MC + AC group compared to the MC group (*p* = 0.01 Fig. 3A and C). Similarly, the immunoreactivity of Occludin increased in the MC + AC group compared to the MC group (*p* = 0.02 Fig. 3B and D). Thus, acetate supplementation prevented the dysregulated expression of tight junction proteins leading to restored gut barrier integrity.

Further, the immunoreactivities of important tight junction proteins Claudin 2 and Occludin were associated with luminal acetate concentration. Luminal acetate concentration negatively correlated with Claudin 2 immunoreactivity (Pearson’s R = – 1.942, *p* < 0.001)(Fig. 3E) whereas occludin immunoreactivity positively correlated with luminal acetate concentration (Pearson’s R = 0.67, *p* < 0.001). (Fig. 3F)

### Acetate supplementation decreased hepatic inflammation induced due to early-life MC-LR exposure

A leaky gut leads to increased translocation of PAMPs and DAMPs in circulation as previously shown by research from our research group and other groups. Increased circulatory levels of various pro-inflammatory molecules cause hepatic inflammation [21, 51, 52]. As previously published by our research group, early life exposure to MC-LR led to MASLD-like inflammation characterized by Kupffer cell and Stellate cell activation [23]. The protein CD68 (Cluster of Differentiation 68) is highly expressed by osteoclasts, monocytic phagocytes, circulating macrophages, and tissue macrophages such as Kupffer cells and microglia [53]. α – Smooth muscle actin (α-SMA) is a marker for the activation of hepatic stellate cells. A subpopulation of activated fibrogenic cells like myofibroblasts and hepatic stellate cells, which are regarded as significant effector cells of tissue fibrogenesis, are identified by the marker α -SMA [54].

**Fig. 4:**
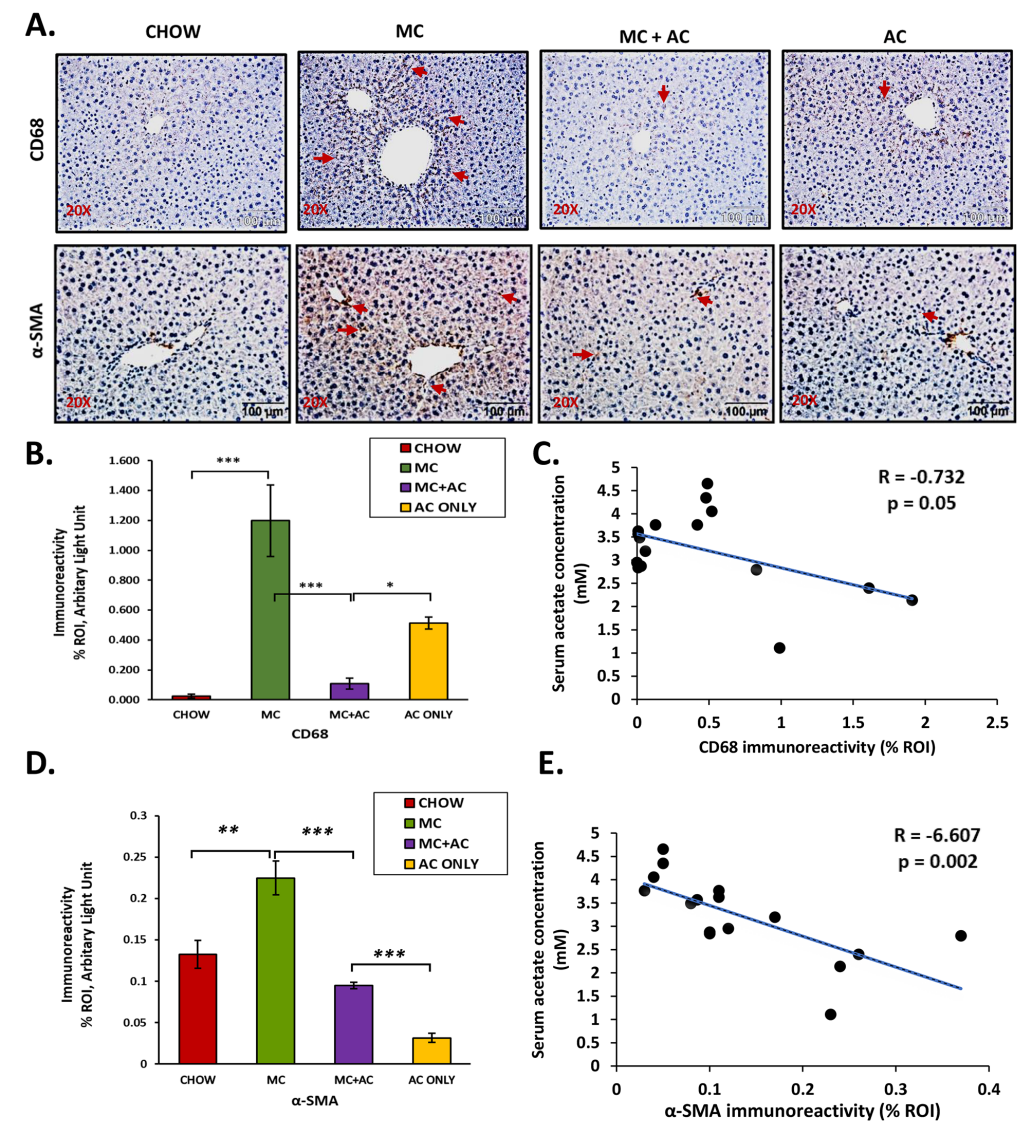
Representative immunohistochemistry images of (**A**) CD68 and (**B**) α-SMA immunoreactivity from CHOW, MC, MC + AC and AC only groups. Morphometric analysis (calculated as %ROI) of (**C**) CD68, and (**D**) α-SMA immunoreactivity where Y-axis represents % positive immunoreactive area (% ROI) (n = 3; mean value taken from three separate microscopic fields). Data were represented as mean ± SEM and statistical significance was tested using unpaired t-test between the two groups (ns – non significant, * *p* < 0.05, ** *p* < 0.01, *** *p* < 0.001), followed by Bonferroni Dunn Post hoc corrections. Correlation plot between serum acetate concentration and liver (**E**) CD68, and (**F**) α-SMA levels

Our current findings corroborated with our previously published results. Immunoreactivity of CD68, a marker for activation of Kupffer cells, was significantly higher in the MC group compared to the CHOW group as measured by immunohistochemical analysis (*p* < 0.001, Fig. 4A and B). Similarly, immunoreactivity of α-SMA, a marker for activation of hepatic stellate cells, was significantly higher in the MC group compared to the CHOW group as measured by immunohistochemical analysis (*p* = 0.005, Fig. 4A and D). The immunoreactivities for both CD68 and α-SMA negatively correlated with serum acetate concentration with Pearson’s R = – 0.73 and – 6.61 respectively (*p* = 0.054 and *p* = 0.002 respectively, Fig. 4C and E).

Acetate supplementation in the MC + AC group ameliorated hepatic inflammatory pathology seen in the MC group. Immunoreactivity against CD68 was significantly lower in liver tissue from mice belonging to the MC + AC group compared to the MC group (*p* < 0.001, Fig. 4A and B). Similarly, immunoreactivity against α-SMA was significantly lower in liver tissue from mice belonging to the MC + AC group compared to the MC group (*p* < 0.001, Fig. 4A and D). This indicated that acetate supplementation in mice lowered the activation of both Kupffer cells and hepatic stellate cells due to early-life MC exposure.

### Acetate supplementation prevented NLRP3 inflammasome activation in response to early-life MC-LR exposure by downregulating NLRP3 protein levels

Previous research from our research group has demonstrated that early-life exposure to MC-LR led to the development of MASLD and this impact was absent in mice lacking the *Nlrp3* gene [23]. Furthermore, we have previously reported that dysbiosis related to MASLD-like conditions after exposure to MC-LR is caused by the activation of the NLRP3 inflammasome [21]. Therefore, NLRP3 inflammasome activation is crucial to MC-LR-induced MASLD caused by early-life gut dysbiosis [55].

**Fig. 5:**
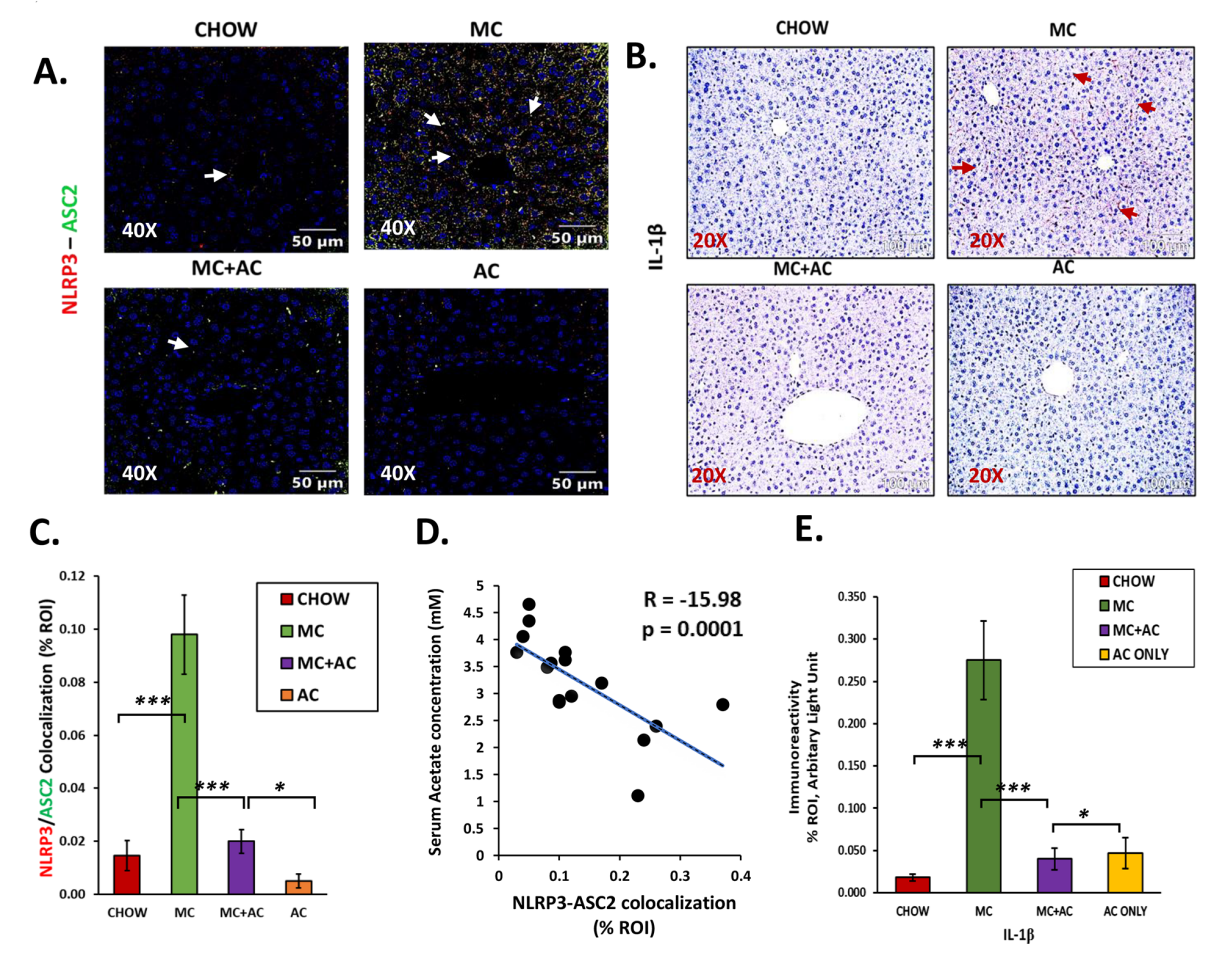
Representative (**A**) immunofluorescence images showing NLRP3 (red) and ASC2 (green) co-localization events and (**B**) immunohistochemistry images of IL-1β immunoreactivity in the liver sections from CHOW, MC, MC + AC and AC only groups. For immunofluorescence images, the liver sections were counterstained with DAPI (blue); all the images were captured in 40× magnification, and immunoreactivity was indicated by white arrows. For immunohistochemistry, all the images were captured in 20× magnification, and immunoreactivity was indicated by red arrows. Morphometric analysis (calculated as %ROI) of (**C**) NLRP3 and ASC2 co-localization events, and (**E**) IL-1β immunoreactivity where Y-axis represents % positive immunoreactive area (% ROI) (n = 3; mean value taken from three separate microscopic fields). Data were represented as mean ± SEM and statistical significance was tested using unpaired t-test between the two groups (ns – non significant, * *p* < 0.05, ** *p* < 0.01, *** *p* < 0.001), followed by Bonferroni–Dunn post hoc corrections. Correlation plot between serum acetate concentration and liver (**D**) NLRP3-ASC2 co-localization events

Toevaluate whether early-life exposure to MC-LR led to NLRP3 inflammasome activation and if acetate treatment prevented this activation, we performed co-immunostaining using antibodies against NLRP3 and ASC2 in liver sections from the four experimental groups to detect the formation of the NLRP3 inflammasome. We also probed for release of IL-1β using immunohistochemistry as it is an effect downstream to NLRP3 inflammasome activation.

We observed a marked increase in co-localization of NLRP3 and ASC2 in the MC group compared to the CHOW group (*p* < 0.001, Fig. 5A and C). There was a corresponding increase in IL-1β immunoreactivity in the MC group compared to the CHOW group (*p* < 0.001, Fig. 5B and E). The increase in co-localization of NLRP3 and ASC2 signified an increased inflammasome formation and activation in the MC group compared to the CHOW group. This increase in inflammasome activation negatively correlated with serum acetate concentrations (Pearson’s R = -15.98, *p* = 0.0001, Fig. 5D). Co-localization of NLRP3 and ASC2 was decreased in the acetate-supplemented MC + AC group as compared to MC only group (*p* = 0.001, Fig. 5A and C). There was a corresponding decrease in IL-1β immunoreactivity in the MC + AC group when compared to the MC group (*p* < 0.001, Fig. 5B and E).

Thus, acetate supplementation indeed decreased the activation of NLRP3 inflammasome and consequent release of the pro-inflammatory cytokine IL-1β in mice that were exposed to MC-LR in early life.

### Acetate supplementation in the microcystin challenge in vivo and in primary kupfferffer cells leads to reduced levels of NLRP3 protein in a GPR43 dependent manner

**Fig. 6:**
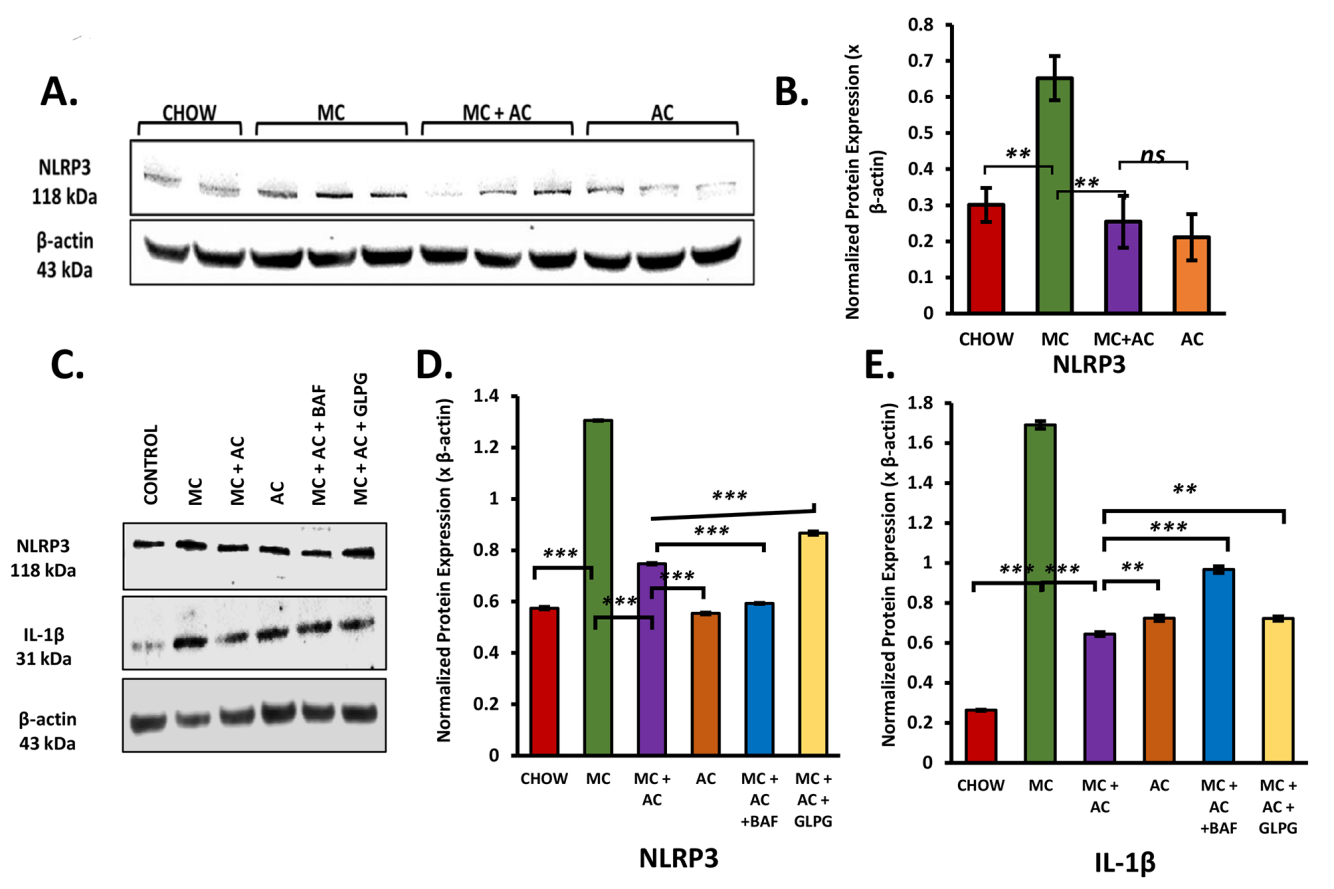
(**A**) Western blot images of NLRP3 and β-actin protein expression levels were obtained from liver tissue extracts from CHOW, MC, MC + AC and AC only groups. Densitometry analyses of (**B**) NLRP3 protein expression normalized against β-actin expression, (**C**) Western blot images of NLRP3, IL-1β and β-actin protein expression levels were obtained from protein extracted from rat Kupffer cells belonging to Control, MC, MC + AC, AC only, MC + AC + Bafilomycin, and MC + AC + GLPG0974 groups. Densitometry analyses of (**D**) NLRP3 and (**E**) IL-1β protein expression normalized against β-actin expression. Data were represented as mean ± SEM and statistical significance was tested using unpaired t-test between the two groups (ns – non significant, * *p* < 0.05, ** *p* < 0.01, *** *p* < 0.001), followed by Bonferroni– Dunn post hoc corrections. Each protein represented as horizontal rows is from the same gel. For Fig. 6A and C, each row is bordered and separated from other rows to indicate that each protein was probed separately either on a fresh blot or on the same blot after stripping them first. Images were acquired using the Biorad ChemiDoc Imaging system with a standard automated exposure and SuperSignal™ West Pico PLUS Chemiluminescent Substrate. 6A and 6C are separate experiments and exposure times for imaging were different. Full length images for 6A and 6C acquired are included as supplementary information

First, we estimated the NLRP3 protein expression in the livers of the experimental mice using the Western Blot method (NLRP3 protein expression was normalized against the expression of β-actin from the same sample). We observed that MC exposure led to an increase in the NLRP3 protein levels compared to the CHOW group (*p* = 0.004, Fig. 6A and B). Acetate supplementation significantly decreased levels of NLRP3 protein (*p* = 0.003, Fig. 6A and B).

Previous literature indicates that acetate treatment in bone marrow-derived macrophages leads to ubiquitin-mediated NLRP3 protein degradation via GPR43 signaling [43]. To confirm the mechanism of whether acetate supplementation in the presence of MC-LR caused NLRP3 autophagy in a GPR43-dependent manner, we designed an in vitro study using rat Kupffer cells. In this study, MC-LR primed rat Kupffer cells (MC) were treated with acetate (MC + AC) in the presence of either GPR43 antagonist GLPG0974 (MC + AC + GLPG) or autophagy inhibitor Bafilomycin A1 (MC + AC + BAF). Chow group was represented by “only cells” control.

We observed that MC-LR challenge in primary rat Kupffer cells (MC) led to an increase in protein levels of NLRP3 (*p* < 0.001, Fig. 6C and D) and acetate supplementation in the presence of MC-LR challenge (MC + AC group) had decreased protein levels of NLRP3 (*p* < 0.001, Fig. 6C and D). There was a corresponding increase in protein levels of IL-1β in MC-treated cells (*p* < 0.001) and a corresponding decrease in the IL-1β levels in acetate-supplemented cells (*p* < 0.001, Fig. 6C and E). These results indicated that MC-LR exposure led to priming of NLRP3 inflammasome and acetate treatment decreased the protein levels of NLRP3 after treatment with MC-LR.

To confirm that the decreased protein levels of NLRP3 protein due to acetate treatment occurred by binding of acetate to its receptor GPR43, we used a GPR43 antagonist – GLPG0974. We expected that the prevention of acetate binding to its receptor GPR43 would reverse the effect acetate had on the protein levels of NLRP3 and its downstream molecule IL-1β. As expected, we observed an increase in protein levels NLRP3 (*p* < 0.001, Fig. 6C and D) and IL-1β (*p* = 0.011, Fig. 6C and E) in the presence of GPR43 receptor antagonist compared to the MC + AC group. This confirmed that acetate in the presence of MC-LR challenge regulated NLRP3 protein levels via its receptor GPR43.

To confirm whether acetate supplementation caused NLRP3 protein degradation via autophagy, we used the autophagy inhibitor Bafilomycin A1 and expected an increase in NLRP3 and IL-1β protein levels in the Bafilomycin A1 treated group compared to MC + AC group. We did observe an increase in IL-1β protein levels in MC + AC + BAF group in comparison to the MC + AC group (*p* < 0.001, Fig. 6C and E). However, we observed a significant decrease in NLRP3 protein levels in Bafilomycin A1 treated group in comparison to the MC + AC group (*p* < 0.001, Fig. 6C and D).

To summarize, MC-LR treatment led to increased protein levels of NLRP3 and IL-1β which were reversed by acetate treatment both in vivo in the liver and in vitro in Kupffer cells. Acetate binding to GPR43 was necessary to decrease protein levels of NLRP3 protein that were increased by MC-LR exposure. Inhibition of autophagy led to a further decrease in NLRP3 protein in vitro. However, an opposite effect was observed in the case of IL-1β.

## Discussion

Previous studies from our research group and others have shown that exposure to MC-LR in adulthood and in early – life leads to gut dysbiosis [8, 21]. We also found that there was a marked decrease in species richness and abundance due to early life exposure to MC-LR using Shannon and Simpson α-diversity indices. Decreased α-diversity is a feature of metabolic conditions like MASLD, type II diabetes, obesity, inflammatory bowel disease, etc. [21, 23, 56–58]. Upon performing Principal Component Analysis using the Bray – Curtis dissimilarity index we observed that the two groups formed separate clusters indicating differences in microbiome signatures between CHOW and MC-treated groups. This indicates that early life exposure to MC-LR leads to a change in the gut microbiome pattern in adolescence. At the family level, this change constituted decreased abundances of *Ruminococcaceae* and *Bacteroidaceae*. *Ruminococcaceae* are a group of strictly anaerobic bacteria that are important producers of SCFA, thus are vital for overall gut health. A decreased abundance of SCFA-producing bacteria has been associated with inflammatory bowel diseases like Crohn’s disease and ulcerative colitis [59, 60]. *Bacteroidaceae* is an important family of bacteria in the gut microbiome that are established early in life [12]. We observed a decrease in the relative abundance of bacteria belonging to the family *Bacteroidaceae* particularly bacteria belonging to the genus *Bacteroides*. The genomes of bacteria belonging to the genus *Bacteroides* code for a vast variety of enzymes that breakdown complex polysaccharides. In humans increased abundance of *Bacteroides* during infancy (approximately 4–8 weeks age in mice) is due to increased consumption of food rich in fibers after weaning. *Bacteroides* spp. are “providers” of the gut ecosystem as they are major producers of SCFA during childhood [61]. Acetate is the most abundant SCFA produced by gut bacteria during childhood and is important for establishing a healthy and stable gut microbiome [61–63].

Since, MC-LR oral exposure immediately after weaning affects the abundance, and richness of bacterial species and causes major alteration in important SCFA producing bacteria it hinders the establishment of a balanced and healthy gut microbiome in adulthood. This led to decreased production of acetate in the gut as measured by acetate assay. As seen in our study, the consequence of this alteration in gut microbiome and its metabolic activities is gut leaching evident from the disruption of the protein levels of tight junction proteins Claudin 2 and Occludin. Moreover, this dysregulation in the protein levels of Claudin 2 and Occludin is associated with luminal acetate concentration in addition to the positive correlation of gut acetate concentrations with abundance of acetate producing species. These results corroborate with previous studies that have described the vital role of SCFAs in maintaining the gut epithelial barrier [64]. However, the mechanism via which acetate modulates the levels of epithelial tight junction proteins is unknown.

Our research group has previously shown that exposure to MC-LR in early life leads to the development of a MASLD like condition characterized by increased activation of Kupffer cells, hepatic stellate cells and release of pro-inflammatory cytokines like IL-1β and TNF-α.Importantly, this potentiation of MASLD like condition was absent in NLRP3 KO mice [23] indicating the central role of NLRP3 in this scenario [23]. The results we obtained also corroborated our previous findings. In our study, we noted that, acetate supplementation prevented the increased activation of Kupffer cells, hepatic stellate cells and release of pro-inflammatory cytokines like IL-1β. Furthermore, acetate supplementation decreased NLRP3 inflammasome assembly and activation. Thus, providing a basis for the therapeutic potential of targeting the NLRP3 inflammasome in the mitigation of toxin associated inflammatory liver diseases. It has been previously shown that acetate upon binding with its receptor GPR43 can cause NLRP3 protein degradation thereby attenuating NLRP3 inflammasome activation in a Ca^2+^ dependent manner [43].

We wanted to elucidate the mechanism by which acetate caused attenuation of NLRP3 inflammasome activation following exposure to MC-LR. We observed that protein levels of NLRP3 in the liver of mice that were exposed to MC-LR received acetate supplementation were lower than those from mice that were exposed to MC-LR without acetate supplementation. The protein level of NLRP3 in mice supplemented with acetate was not significantly different from that in unexposed mice i.e., the CHOW group as well as the mice that received only acetate supplementation i.e., the AC group. We theorized based on a literature review that the decreased NLRP3 levels in MC + AC group was due to acetate binding its receptor GPR43 and its downstream effects [43]. So, we performed the in vitro experiments using rat primary Kupffer cells where we supplemented the cell culture medium with acetate and treated the mice to MC-LR. One of the test groups were first treated with a GPR43 antagonist before acetate supplementation. It was observed that the levels of NLRP3 protein were elevated in the presence of GPR43 antagonist suggesting that binding of acetate to its receptor GPR43 is essential for reducing NLRP3 protein levels in MC treated cells. Further, the protein levels of IL-1β were also elevated suggesting that acetate – GPR43 signaling was important for NLRP3 inflammasome assembly and downstream IL-1β release.

Next, based on literature review we hypothesized that acetate – GPR43 signaling led to NLRP3 protein degradation by autophagy when treated with MC-LR [43]. So, we used Bafilomycin A1 an inhibitor of autophagy before supplementing primary rat Kupffer cells with acetate followed by exposure to MC-LR. We observed that the protein levels of IL-1β elevated as expected. Surprisingly, the protein levels of NLRP3 further decreased when the cells were treated with an inhibitor of autophagy.

NLRP3 protein is post-translationally regulated in many ways. This includes inactivation by ubiquitination, ubiquitination-mediated autophagy, ubiquitination mediated proteasomal degradation, phosphorylation, acetylation, nitrosylation, and SUMOylation [65]. NLRP3 protein degradation can occur via autophagy triggered by K63 polyubiquitination of NLRP3 protein and subsequent interaction with autophagy adaptor p62 [66] or via proteasomal degradation triggered by K63 and K48 linked ubiquitination by E3 ubiquitin ligases ring finger protein RNF125 and Casitas B-lineage lymphoma proto-oncogene-b Cbl-b [67]. Since the Bafilomycin A1 inhibits autophagy [68] and not proteasomal degradation, acetate may cause NLRP3 protein degradation via proteasomal degradation and not autophagy. Further, experiments using inhibitors of E3 ubiquitin ligases RNF125 and Cbl-b are necessary to verify this possible mechanism. Also, acetate – GPR43 signaling may prevent the priming step of NLRP3 inflammasome activation that includes the increased mRNA expression of NLRP3, pro-IL-1β and pro-IL-18 in response to TLR4 activation or TNF signaling [69]. Further, studies need to be carried out to link acetate – GPR43 signaling with NLRP3 inflammasome regulation and degradation.

**Fig. 7:**
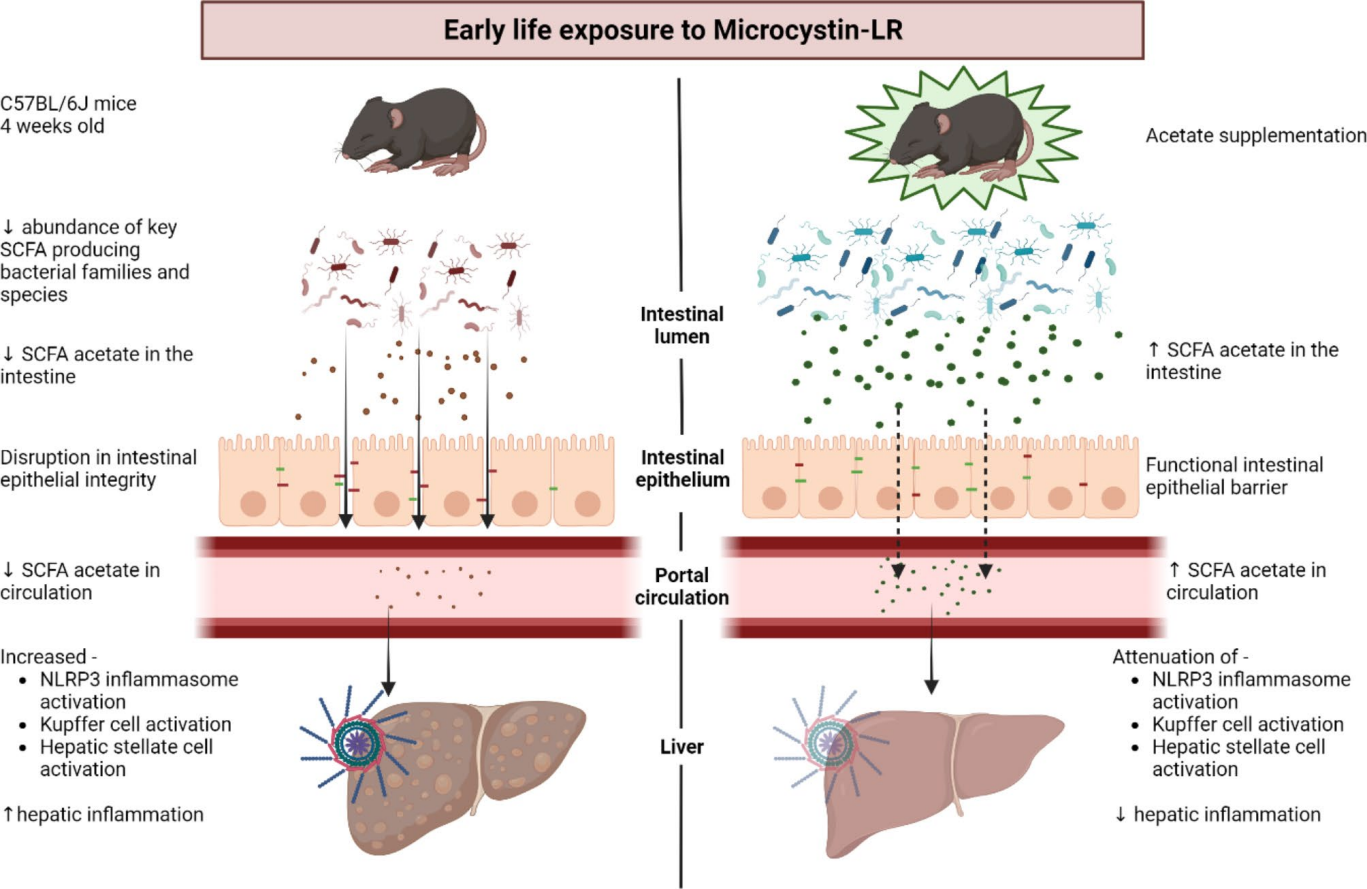
Graphical representation of the mechanistic endpoints describing the role acetate in early life microcystin exposure. This figure was prepared by authors for this publication using BioRender Software

In conclusion, this study highlights the importance of a healthy gut microbiome and its metabolites like SCFAs in alleviating inflammation caused due to exposure to cyanotoxin MC-LR. This study along with previous publications establishes the central role of NLRP3 inflammasome priming and activation in promoting hepatic inflammation due to exposure to MC-LR in early life. It draws attention towards gut dysbiosis and NLRP3 inflammasome as therapeutic targets for treating environment-linked fatty liver diseases and other inflammatory disorders. Lastly, it tries to elucidate the mechanism by which gut metabolites like SCFA particularly acetate can modulate inflammation via the Gut – Liver Axis (Fig. 7).

### Electronic supplementary material

Below is the link to the electronic supplementary material.

Supplementary Material 1

## Data Availability

All data presented in this study are available on request from the corresponding author.

## References

[1] Lu J, Struewing I, Wymer L, Tettenhorst DR, Shoemaker J, Allen J (2020). Use of qPCR and RT-qPCR for monitoring variations of microcystin producers and as an early warning system to predict toxin production in an Ohio inland lake. Water Res.

[2] Cheung MY, Liang S, Lee J (2013). Toxin-producing cyanobacteria in freshwater: a review of the problems, impact on drinking water safety, and efforts for protecting public health. J Microbiol.

[3] Kozdeba M, Borowczyk J, Zimolag E, Wasylewski M, Dziga D, Madeja Z (2014). Microcystin-LR affects properties of human epidermal skin cells crucial for regenerative processes. Toxicon.

[4] Backer LC, McNeel SV, Barber T, Kirkpatrick B, Williams C, Irvin M (2010). Recreational exposure to microcystins during algal blooms in two California lakes. Toxicon.

[5] Schaefer AM, Yrastorza L, Stockley N, Harvey K, Harris N, Grady R (2020). Exposure to microcystin among coastal residents during a cyanobacteria bloom in Florida. Harmful Algae.

[6] Selevan SG, Kimmel CA, Mendola P (2000). Identifying critical windows of exposure for children’s health. Environ Health Perspect.

[7] Beath SV (2003). Hepatic function and physiology in the newborn. Semin Neonatol.

[8] Saha P, Bose D, Stebliankin V, Cickovski T, Seth RK, Porter DE (2022). Prior exposure to microcystin alters host gut resistome and is associated with dysregulated immune homeostasis in translatable mouse models. Sci Rep.

[9] Ayeni KI, Berry D, Wisgrill L, Warth B, Ezekiel CN (2022). Early-life chemical exposome and gut microbiome development: African research perspectives within a global environmental health context. Trends Microbiol.

[10] Scott NA, Andrusaite A, Andersen P, Lawson M, Alcon-Giner C, Leclaire C, et al. Antibiotics induce sustained dysregulation of intestinal T cell immunity by perturbing macrophage homeostasis. Sci Transl Med. 2018;10(464). 10.1126/scitranslmed.aao4755. PubMed PMID: 30355800; PubMed Central PMCID: PMC6548564. 10.1126/scitranslmed.aao4755 PMC6548564 30355800

[11] Dai J, Yang X, Yuan Y, Jia Y, Liu G, Lin N (2020). Toxicity, gut microbiota and metabolome effects after copper exposure during early life in SD rats. Toxicology.

[12] Zafar H, Saier MH (2021). Jr. Gut Bacteroides species in health and Disease. Gut Microbes.

[13] Thorburn AN, McKenzie CI, Shen S, Stanley D, Macia L, Mason LJ (2015). Evidence that Asthma is a developmental origin Disease influenced by maternal diet and bacterial metabolites. Nat Commun.

[14] Holota Y, Dovbynchuk T, Kaji I, Vareniuk I, Dzyubenko N, Chervinska T (2019). The long-term consequences of antibiotic therapy: role of colonic short-chain fatty acids (SCFA) system and intestinal barrier integrity. PLoS ONE.

[15] Bokulich NA, Chung J, Battaglia T, Henderson N, Jay M, Li H (2016). Antibiotics, birth mode, and diet shape microbiome maturation during early life. Sci Transl Med.

[16] Pammi M, Cope J, Tarr PI, Warner BB, Morrow AL, Mai V (2017). Intestinal dysbiosis in preterm infants preceding necrotizing enterocolitis: a systematic review and meta-analysis. Microbiome.

[17] Alsharairi NA. The Role of Short-Chain Fatty Acids in Mediating Very Low-Calorie Ketogenic Diet-Infant Gut Microbiota Relationships and Its Therapeutic Potential in Obesity. Nutrients. 2021;13(11). Epub 20211021. 10.3390/nu13113702. PubMed PMID: 34835958; PubMed Central PMCID: PMC8624546. 10.3390/nu13113702 PMC8624546 34835958

[18] Gur TL, Shay L, Palkar AV, Fisher S, Varaljay VA, Dowd S (2017). Prenatal stress affects placental cytokines and neurotrophins, commensal microbes, and anxiety-like behavior in adult female offspring. Brain Behav Immun.

[19] Mondal A, Saha P, Bose D, Chatterjee S, Seth RK, Xiao S (2021). Environmental microcystin exposure in underlying NAFLD-induced exacerbation of neuroinflammation, blood-brain barrier dysfunction, and neurodegeneration are NLRP3 and S100B dependent. Toxicology.

[20] Sarkar S, Alhasson F, Kimono D, Albadrani M, Seth RK, Xiao S (2020). Microcystin exposure worsens nonalcoholic fatty Liver Disease associated ectopic glomerular toxicity via NOX-2-MIR21 axis. Environ Toxicol Pharmacol.

[21] Sarkar S, Kimono D, Albadrani M, Seth RK, Busbee P, Alghetaa H (2019). Environmental microcystin targets the microbiome and increases the risk of intestinal inflammatory pathology via NOX2 in underlying murine model of nonalcoholic fatty Liver Disease. Sci Rep.

[22] European Association for the Study of the Liver (2023). Electronic address glo, American Association for the Study of Liver D, Latin American Association for the study of the L. A call for unity: the path towards a more precise and patient-centric nomenclature for NAFLD. J Hepatol.

[23] Al-Badrani M, Saha P, Mondal A, Seth RK, Sarkar S, Kimono D (2020). Early microcystin-LR exposure-linked inflammasome activation in mice causes development of fatty Liver Disease and insulin resistance. Environ Toxicol Pharmacol.

[24] He Y, Hara H, Nunez G (2016). Mechanism and regulation of NLRP3 inflammasome activation. Trends Biochem Sci.

[25] Bauernfeind FG, Horvath G, Stutz A, Alnemri ES, MacDonald K, Speert D (2009). Cutting edge: NF-kappaB activating pattern recognition and cytokine receptors license NLRP3 inflammasome activation by regulating NLRP3 expression. J Immunol.

[26] Lin KM, Hu W, Troutman TD, Jennings M, Brewer T, Li X (2014). IRAK-1 bypasses priming and directly links TLRs to rapid NLRP3 inflammasome activation. Proc Natl Acad Sci U S A.

[27] Xing Y, Yao X, Li H, Xue G, Guo Q, Yang G (2017). Cutting Edge: TRAF6 mediates TLR/IL-1R Signaling-Induced Nontranscriptional Priming of the NLRP3 inflammasome. J Immunol.

[28] Murakami T, Ockinger J, Yu J, Byles V, McColl A, Hofer AM (2012). Critical role for calcium mobilization in activation of the NLRP3 inflammasome. Proc Natl Acad Sci U S A.

[29] Munoz-Planillo R, Kuffa P, Martinez-Colon G, Smith BL, Rajendiran TM, Nunez G (2013). K(+) efflux is the common trigger of NLRP3 inflammasome activation by bacterial toxins and particulate matter. Immunity.

[30] Zhou R, Yazdi AS, Menu P, Tschopp J. A role for mitochondria in NLRP3 inflammasome activation. Nature. 2011;469(7329):221-5. Epub 20101201. 10.1038/nature09663. PubMed PMID: 21124315. 10.1038/nature09663 21124315

[31] Schorn C, Frey B, Lauber K, Janko C, Strysio M, Keppeler H (2011). Sodium overload and water influx activate the NALP3 inflammasome. J Biol Chem.

[32] He WT, Wan H, Hu L, Chen P, Wang X, Huang Z (2015). Gasdermin D is an executor of pyroptosis and required for interleukin-1beta secretion. Cell Res.

[33] Yang Y, Gong P, Long X, Jiang Y, Ye M, Tao S et al. Microcystin-LR Induces and Aggravates Colitis through NLRP3 Inflammasome-Mediated Pyroptosis in Mice. Toxins (Basel). 2023;15(7). Epub 20230706. 10.3390/toxins15070447. PubMed PMID: 37505716. 10.3390/toxins15070447 PMC10467093 37505716

[34] Zhang Y, Zhu P, Wu X, Yuan T, Su Z, Chen S (2021). Microcystin-LR induces NLRP3 inflammasome activation via FOXO1 phosphorylation, resulting in Interleukin-1beta secretion and Pyroptosis in hepatocytes. Toxicol Sci.

[35] Guilin Z, Pengyu Z, Wei L, Fengqi H, Chen F, Yu Y (2020). Reduction of gut microbial diversity and short chain fatty acids in BALB/c mice exposure to microcystin-LR. Ecotoxicology.

[36] Lee J, Lee S, Mayta A, Mrdjen I, Weghorst C, Knobloch T (2020). Microcystis toxin-mediated Tumor promotion and toxicity lead to shifts in mouse gut microbiome. Ecotoxicol Environ Saf.

[37] Gao Y, Davis B, Zhu W, Zheng N, Meng D, Walker WA (2021). Short-chain fatty acid butyrate, a breast milk metabolite, enhances immature intestinal barrier function genes in response to inflammation in vitro and in vivo. Am J Physiol Gastrointest Liver Physiol.

[38] Zheng N, Gao Y, Zhu W, Meng D, Walker WA (2020). Short chain fatty acids produced by colonizing intestinal commensal bacterial interaction with expressed breast milk are anti-inflammatory in human immature enterocytes. PLoS ONE.

[39] Smith PM, Howitt MR, Panikov N, Michaud M, Gallini CA, Bohlooly YM (2013). The microbial metabolites, short-chain fatty acids, regulate colonic Treg cell homeostasis. Science.

[40] Furusawa Y, Obata Y, Fukuda S, Endo TA, Nakato G, Takahashi D (2013). Commensal microbe-derived butyrate induces the differentiation of colonic regulatory T cells. Nature.

[41] Trompette A, Gollwitzer ES, Yadava K, Sichelstiel AK, Sprenger N, Ngom-Bru C (2014). Gut microbiota metabolism of dietary fiber influences allergic airway Disease and hematopoiesis. Nat Med.

[42] Sun J, Furio L, Mecheri R, van der Does AM, Lundeberg E, Saveanu L (2015). Pancreatic beta-cells limit Autoimmune Diabetes via an immunoregulatory antimicrobial peptide expressed under the influence of the gut microbiota. Immunity.

[43] Xu M, Jiang Z, Wang C, Li N, Bo L, Zha Y (2019). Acetate attenuates inflammasome activation through GPR43-mediated ca(2+)-dependent NLRP3 ubiquitination. Exp Mol Med.

[44] Caetano-Silva ME, Rund L, Hutchinson NT, Woods JA, Steelman AJ, Johnson RW (2023). Inhibition of inflammatory microglia by dietary fiber and short-chain fatty acids. Sci Rep.

[45] Li W, Deng M, Gong J, Hou Y, Zhao L. Bidirectional Regulation of Sodium Acetate on Macrophage Activity and Its Role in Lipid Metabolism of Hepatocytes. Int J Mol Sci. 2023;24(6). Epub 20230314. 10.3390/ijms24065536. PubMed PMID: 36982619; PubMed Central PMCID: PMC10051801. 10.3390/ijms24065536 PMC10051801 36982619

[46] Parada Venegas D, De la Fuente MK, Landskron G, Gonzalez MJ, Quera R, Dijkstra G (2019). Short chain fatty acids (SCFAs)-Mediated gut epithelial and Immune Regulation and its relevance for inflammatory Bowel Diseases. Front Immunol.

[47] Yamamoto A, Tagawa Y, Yoshimori T, Moriyama Y, Masaki R, Tashiro Y (1998). Bafilomycin A1 prevents maturation of autophagic vacuoles by inhibiting fusion between autophagosomes and lysosomes in rat hepatoma cell line, H-4-II-E cells. Cell Struct Funct.

[48] Chen Y, Lear TB, Evankovich JW, Larsen MB, Lin B, Alfaras I (2021). A high-throughput screen for TMPRSS2 expression identifies FDA-approved compounds that can limit SARS-CoV-2 entry. Nat Commun.

[49] Pizzonero M, Dupont S, Babel M, Beaumont S, Bienvenu N, Blanque R (2014). Discovery and optimization of an azetidine chemical series as a free fatty acid receptor 2 (FFA2) antagonist: from hit to clinic. J Med Chem.

[50] Saha P, Skidmore PT, Holland LA, Mondal A, Bose D, Seth RK et al. Andrographolide Attenuates Gut-Brain-Axis Associated Pathology in Gulf War Illness by Modulating Bacteriome-Virome Associated Inflammation and Microglia-Neuron Proinflammatory Crosstalk. Brain Sci. 2021;11(7). Epub 20210709. 10.3390/brainsci11070905. PubMed PMID: 34356139; PubMed Central PMCID: PMC8304847. 10.3390/brainsci11070905 PMC8304847 34356139

[51] Sarkar S, Saha P, Seth RK, Mondal A, Bose D, Kimono D (2020). Higher intestinal and circulatory lactate associated NOX2 activation leads to an ectopic fibrotic pathology following microcystin co-exposure in murine fatty Liver Disease. Comp Biochem Physiol C Toxicol Pharmacol.

[52] Albadrani M, Seth RK, Sarkar S, Kimono D, Mondal A, Bose D (2019). Exogenous PP2A inhibitor exacerbates the progression of nonalcoholic fatty Liver Disease via NOX2-dependent activation of miR21. Am J Physiol Gastrointest Liver Physiol.

[53] Chistiakov DA, Killingsworth MC, Myasoedova VA, Orekhov AN, Bobryshev YV (2017). CD68/macrosialin: not just a histochemical marker. Lab Invest.

[54] Zhao W, Wang X, Sun KH, Zhou L (2018). Alpha-smooth muscle actin is not a marker of fibrogenic cell activity in skeletal muscle fibrosis. PLoS ONE.

[55] Blevins HM, Xu Y, Biby S, Zhang S (2022). The NLRP3 inflammasome pathway: a review of mechanisms and inhibitors for the treatment of Inflammatory Diseases. Front Aging Neurosci.

[56] Hoang T, Kim M, Park JW, Jeong SY, Lee J, Shin A (2023). Dysbiotic microbiome variation in Colorectal cancer patients is linked to lifestyles and metabolic Diseases. BMC Microbiol.

[57] Du Y, Li X, Su C, Xi M, Zhang X, Jiang Z (2020). Butyrate protects against high-fat diet-induced Atherosclerosis via up-regulating ABCA1 expression in apolipoprotein E-deficiency mice. Br J Pharmacol.

[58] Sathkumara HD, Eaton JL, Field MA, Govan BL, Ketheesan N, Kupz A (2021). A murine model of tuberculosis/type 2 Diabetes comorbidity for investigating the microbiome, metabolome and associated immune parameters. Anim Model Exp Med.

[59] Khanna S, Raffals LE (2017). The Microbiome in Crohn’s Disease: Role in Pathogenesis and Role of Microbiome replacement therapies. Gastroenterol Clin North Am.

[60] Shen ZH, Zhu CX, Quan YS, Yang ZY, Wu S, Luo WW (2018). Relationship between intestinal microbiota and ulcerative Colitis: mechanisms and clinical application of probiotics and fecal microbiota transplantation. World J Gastroenterol.

[61] Rodriguez JM, Murphy K, Stanton C, Ross RP, Kober OI, Juge N (2015). The composition of the gut microbiota throughout life, with an emphasis on early life. Microb Ecol Health Dis.

[62] Laursen MF, Bahl MI, Michaelsen KF, Licht TR (2017). First Foods and Gut microbes. Front Microbiol.

[63] Rinninella E, Cintoni M, Raoul P, Lopetuso LR, Scaldaferri F, Pulcini G et al. Food Components and Dietary Habits: Keys for a Healthy Gut Microbiota Composition. Nutrients. 2019;11(10). Epub 20191007. 10.3390/nu11102393. PubMed PMID: 31591348; PubMed Central PMCID: PMC6835969. 10.3390/nu11102393 PMC6835969 31591348

[64] Suzuki T, Yoshida S, Hara H (2008). Physiological concentrations of short-chain fatty acids immediately suppress colonic epithelial permeability. Br J Nutr.

[65] Paik S, Kim JK, Silwal P, Sasakawa C, Jo EK (2021). An update on the regulatory mechanisms of NLRP3 inflammasome activation. Cell Mol Immunol.

[66] Zhou Z, Zhu X, Yin R, Liu T, Yang S, Zhou L (2020). K63 ubiquitin chains target NLRP3 inflammasome for autophagic degradation in ox-LDL-stimulated THP-1 macrophages. Aging.

[67] Tang J, Tu S, Lin G, Guo H, Yan C, Liu Q, et al. Sequential ubiquitination of NLRP3 by RNF125 and Cbl-b limits inflammasome activation and endotoxemia. J Exp Med. 2020;217(4). 10.1084/jem.20182091. PubMed PMID: 31999304; PubMed Central PMCID: PMC7144527. 10.1084/jem.20182091 PMC7144527 31999304

[68] Mauvezin C, Neufeld TP (2015). Bafilomycin A1 disrupts autophagic flux by inhibiting both V-ATPase-dependent acidification and Ca-P60A/SERCA-dependent autophagosome-lysosome fusion. Autophagy.

[69] McKee CM, Coll RC (2020). NLRP3 inflammasome priming: a riddle wrapped in a mystery inside an enigma. J Leukoc Biol.

