# Supplementary material for: Host microbiome associated low intestinal acetate correlates with progressive NLRP3-dependent hepatic-immunotoxicity in early life microcystin-LR exposure
Source: BMC Pharmacol Toxicol. 2023 Dec 13;24:78. doi: 10.1186/s40360-023-00721-7 (PMC10720243; doi:10.1186/s40360-023-00721-7)
Supplement: Supplementary file 1 — Supplementary Material 1 [file 40360_2023_721_MOESM1_ESM.pdf]

In vivo Liver Protein
Western Blots – full blot images

Order from left to right:-

1. Ladder
2. CHOW
3. CHOW
4. MC
5. MC
6. MC
7. MC + AC
8. MC + AC
9. MC + AC
10. AC
11. AC
12. AC

NLRP3 118 kDa

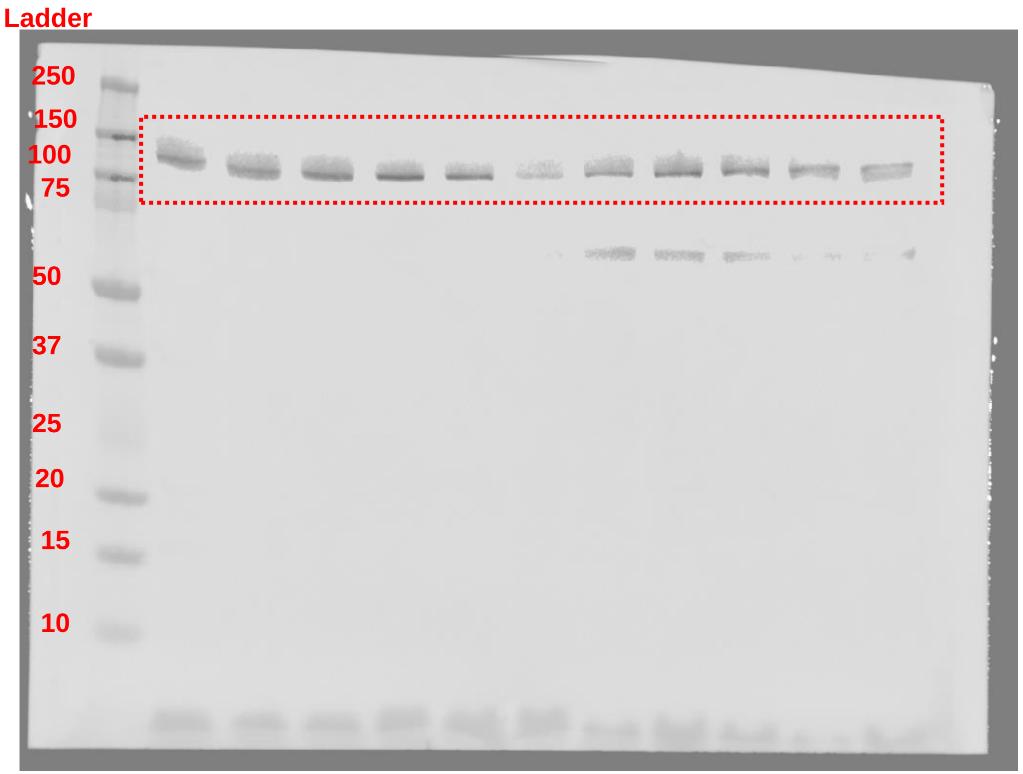

β-actin 43kDa

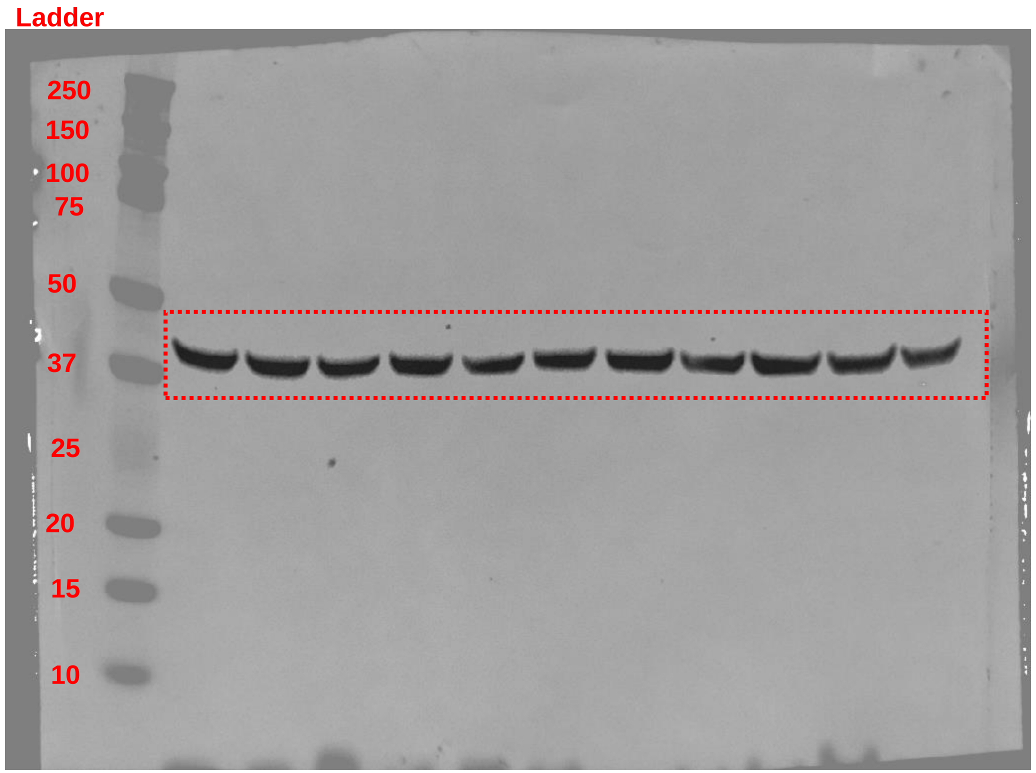

In vitro Protein extracts
Western Blots – full blot images

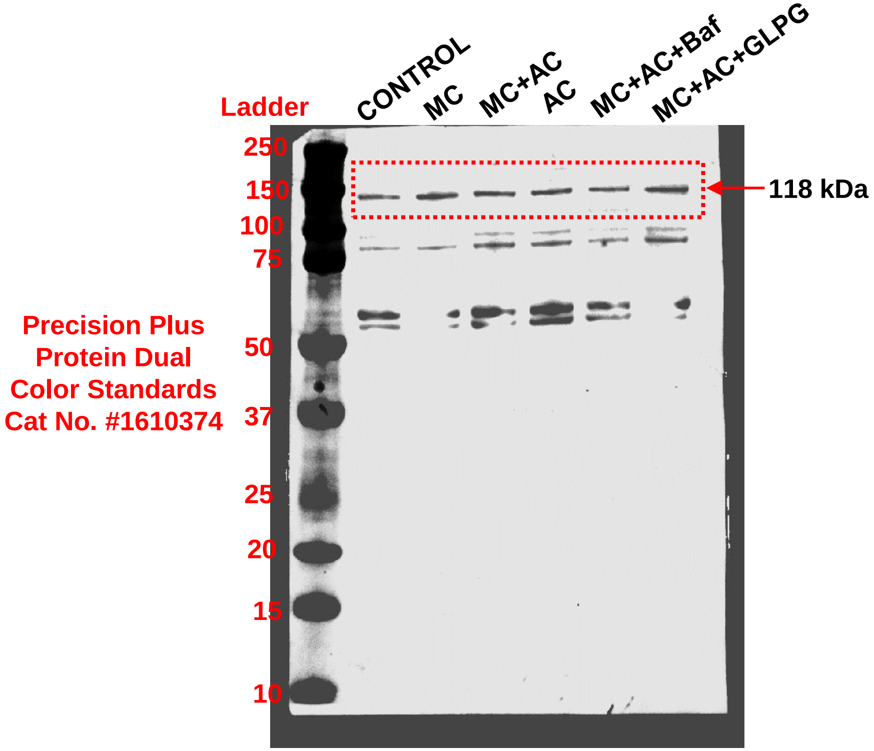

NLRP3

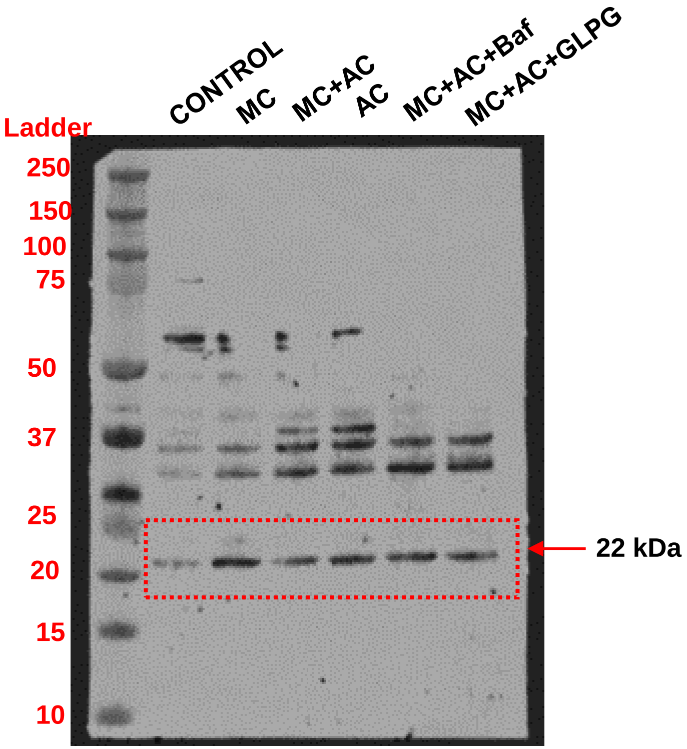

IL-1β

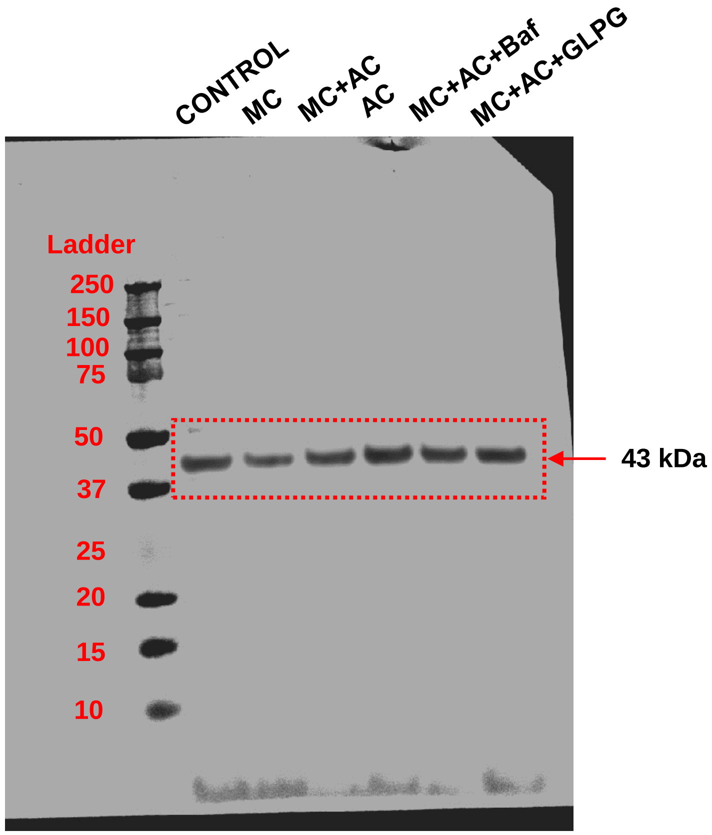

β-Actin
